# Editorial: Arteriogenesis and Collateral Remodelling in Ischaemic Disease

**DOI:** 10.3389/fcvm.2022.916218

**Published:** 2022-06-15

**Authors:** Junxi Wu, Dongxing Zhu, Susan Currie

**Affiliations:** ^1^Department of Biomedical Engineering, University of Strathclyde, Glasgow, United Kingdom; ^2^Guangdong Key Laboratory of Vascular Diseases, State Key Laboratory of Respiratory Disease, School of Basic Medical Sciences, The Second Affiliated Hospital, Guangzhou Institute of Cardiovascular Disease, Guangzhou Medical University, Guangzhou, China; ^3^Strathclyde Institute of Pharmacy and Biomedical Sciences, University of Strathclyde, Glasgow, United Kingdom

**Keywords:** ischaemia, reperfusion, arteriogenesis, collateral artery, vascular remodeling

Development of atherosclerosis leads to narrowing/blockage of large and medium sized arteries and subsequent restriction of blood supply to downstream tissues. Ischaemic disease, including ischaemic heart disease and critical limb ischaemia, presents a serious medical and economic burden to aging societies (1). Reopening or replacing the diseased artery (by percutaneous intervention with stenting or by vascular bypass surgery) are effective therapeutic strategies to restore perfusion in the ischaemic tissue. However, these invasive interventions are typically employed at a late-stage of disease when critical ischaemia has been well established. Notably, there is a large group of patients who are not eligible/suitable for surgical intervention, either because of the complex vascular pathology or lack of suitable autologous vascular grafts. Crucially, any validated alternative interventions for ischaemic disease are still lacking (2).

The concept of therapeutic angiogenesis was developed in this context with the aim to promote reperfusion in the ischaemic tissue *via* stimulation of angiogenesis (3). A range of therapeutic approaches have been explored to encourage angiogenesis around and in the ischaemic tissue that includes delivering proangiogenic factors, proangiogenic gene therapies and stem cell-based therapies (3–6). Unfortunately, the clinical therapeutic benefit of any of these approaches is marginal and inconclusive. There is a substantial amount of investigation and discussion around the technological issues of these therapeutic approaches including the delivery, retention and viability of transplanted cells (7, 8), and the side effects of gene therapy (e.g., inflammation, off-target angiogenesis, plaque destabilization and thrombosis) (2). It is yet to be determined whether improvement of therapeutic technologies may further improve the efficacy of therapeutic angiogenesis.

To date, the majority of research in this area focuses on stimulation of angiogenesis, whereas the importance of arteriogenesis (or collateral artery growth) on effective reperfusion is somewhat overlooked. A key fact is that the vascular diameter is a determinant of the vessel's ability to conduct blood. According to Poiseuille's Law, Q = (Δ*P*π^4^)/(8μ*L*), the ability of an artery to conduct blood flow is proportional to the 4th power of its radius. That is to say, it will require 1.6 billion new microvessels (at diameter of 10 μm) to replace a blocked artery (at a diameter of 2 mm). In this sense, angiogenesis on its own is very unlikely to re-establish the urgently needed blood supply to ischaemic tissue.

By contrast, the vascular network does reserve capability to grow collateral arteries to boost blood supply in response to ischaemia or increased metabolic demand. In fact, some therapeutic angiogenesis studies have reported significant collateral growth along with angiogenesis, revealing that some proangiogenic factors also have direct effect on arteriogenesis (3–6). Nevertheless, the availability of focused research on mechanisms of arteriogenesis and collateralisation are insufficient despite the great potential of this approach in therapeutics.

In the Research Topic ‘*Arteriogenesis and Collateral Remodelling in Ischaemic Disease*', Wu et al. report an alternative strategy to enhance angiogenesis which does not improve reperfusion in a mouse hindlimb ischaemia model. In this study, the correlation between the rapid reperfusion and collateral artery growth in the first week suggests arteriogenesis plays a critical role in early post-ischaemia reperfusion. This is in line with the evidence that following inhibition of arteriogenesis (e.g., by CD44 knockout) in a similar animal model, there was significant suppression of collateral growth and reperfusion by 1 week (9). Further research is needed to understand the role of arteriogenesis as a quick responding mechanism to relieve acute ischaemia and the potential it may have as a therapeutic target (10). In the clinical sector, Shao et al. report retrospective evidence that visceral obesity correlates with poor coronary collateralisation and poor prognosis. This reassures the clinical importance of collateralisation in ischaemic heart disease. The fact that collateralisation is regulated by the patients pathological condition (visceral obesity in this case) suggests that it could serve as a valid therapeutic target.

Monocyte recruitment is a well-recognized mechanism to initiate arteriogenesis (11). However, the detailed mechanism of collateral growth is still not well understood. The study by Andraska et al. provide new insights to vascular matrix remodeling following monocyte recruitment. The counter balanced elastolysis and elastogenesis plays a critical role in the expansion of diameter of the collateral artery. A more comprehensive picture of matrix remodeling in arteriogenesis is summarized in a review by Kulkarni et al.. It is becoming clear that arteriogenesis and atherosclerosis share some common underlying pathways in matrix remodeling, and the outward growth during arteriogenesis leads to a weakened vascular matrix and increases the risk of developing an aneurysm. This dilemma makes it very difficult to develop effective medical interventions to stimulate arteriogenesis.

Jiang et al. report that intramuscular injection of autologous peripheral blood mononuclear cells to no-option critical limb ischemia patients improves the quality of life and reduces the major amputation rate up to 3 years. Although the exact mechanism of this cell-based therapy is unclear, it is possible that local administration of mononuclear cells may mimic the effect of recruitment of monocytes to collateral arteries. There is no doubt that the positive clinical outcome reported in this study will stimulate follow-up studies to reveal the underlining mechanism and to refine the clinical therapeutic protocol.

Finally, the work by Silva et al. further extends our understanding of arteriogenesis from physical enlargement to dynamic functional adaptation. It reveals that the temporal denervation and reinnervation of the arterial wall are closely coupled with smooth muscle cell proliferation and subsequent functional maturation. This opens up the question on how maturation of the arterial wall following arteriogenesis may affect downstream perfusion.

The accumulating evidence of the importance of arteriogenesis in ischaemic disease is raising the awareness of its value as a therapeutic target. Meanwhile, powerful research tools are still essential to allow accurate assessment of the collateral growth and the efficacy of novel treatments in ischaemic reperfusion, including high resolution 3D imaging, quantification and analysis of collateral size, shape, functionality, networking, hemodynamics and downstream perfusion.

## Author Contributions

JW, DZ, and SC: developing/editing the Research Topic and writing/revising the editorial manuscript. All authors contributed to the article and approved the submitted version.

## Conflict of Interest

The authors declare that the research was conducted in the absence of any commercial or financial relationships that could be construed as a potential conflict of interest.

## Publisher's Note

All claims expressed in this article are solely those of the authors and do not necessarily represent those of their affiliated organizations, or those of the publisher, the editors and the reviewers. Any product that may be evaluated in this article, or claim that may be made by its manufacturer, is not guaranteed or endorsed by the publisher.
